# Supplementary material for: Cell-free Chromatin Immunoprecipitation to detect molecular pathways in Physiological and Disease States
Source: bioRxiv. 2023 Jan 25:2023.01.24.525414. Preprint. [Version 1] doi: 10.1101/2023.01.24.525414 (PMC9928031; doi:10.1101/2023.01.24.525414)
Supplement: Supplement 1 [file NIHPP2023.01.24.525414v1-supplement-1.pdf]

bioRxiv preprint doi: https://doi.org/10.1101/2023.01.24.525414; this version posted January 25, 2023. The copyright holder for this preprint (which was not certified by peer review) is the author/funder, who has granted bioRxiv a license to display the preprint in perpetuity. It is made available under aCC-BY 4.0 International license.

Supplementary Figure Legend

Suppl Figure 1. Optimization of cfChIP-seq. **(A)** Saturation curve of individual samples for input DNA and chromatin (H3K36Me3, H3K4Me1, H3K4Me2 and H3K4Me3) profile. **(B)** Distribution of cfChIP-seq signals on genes around the TSS and TES for with and without input normalization.

Suppl. Figure 2. Comparable frequency distribution of chromatin profile among blood processing conditions. **(A)** Frequency distribution of H3K4me1, H3K4me2, H3K36me3, and IgG signals around the TSS in different processing conditions: E16, E3, S16, S3. **(B)** Frequency distribution of H3K36me3 across gene bodies in the different processing conditions.

Suppl. Figure 3. Profiles of the cell-free chromatin. **(A)** Length distribution of sequenced DNA fragments for heart transplant patients. **(B)** Frequency distribution for K4Me3 around the TSS site for heart transplant patients.

Suppl. Figure 4. ChIP-seq tissue-specific signatures in different blood processing conditions. **(A)** Comparison of tissue-specific signatures among the four processing conditions (E16, E3, S16, S3). **(B)** The proportion of blood cells signatures between different conditions.

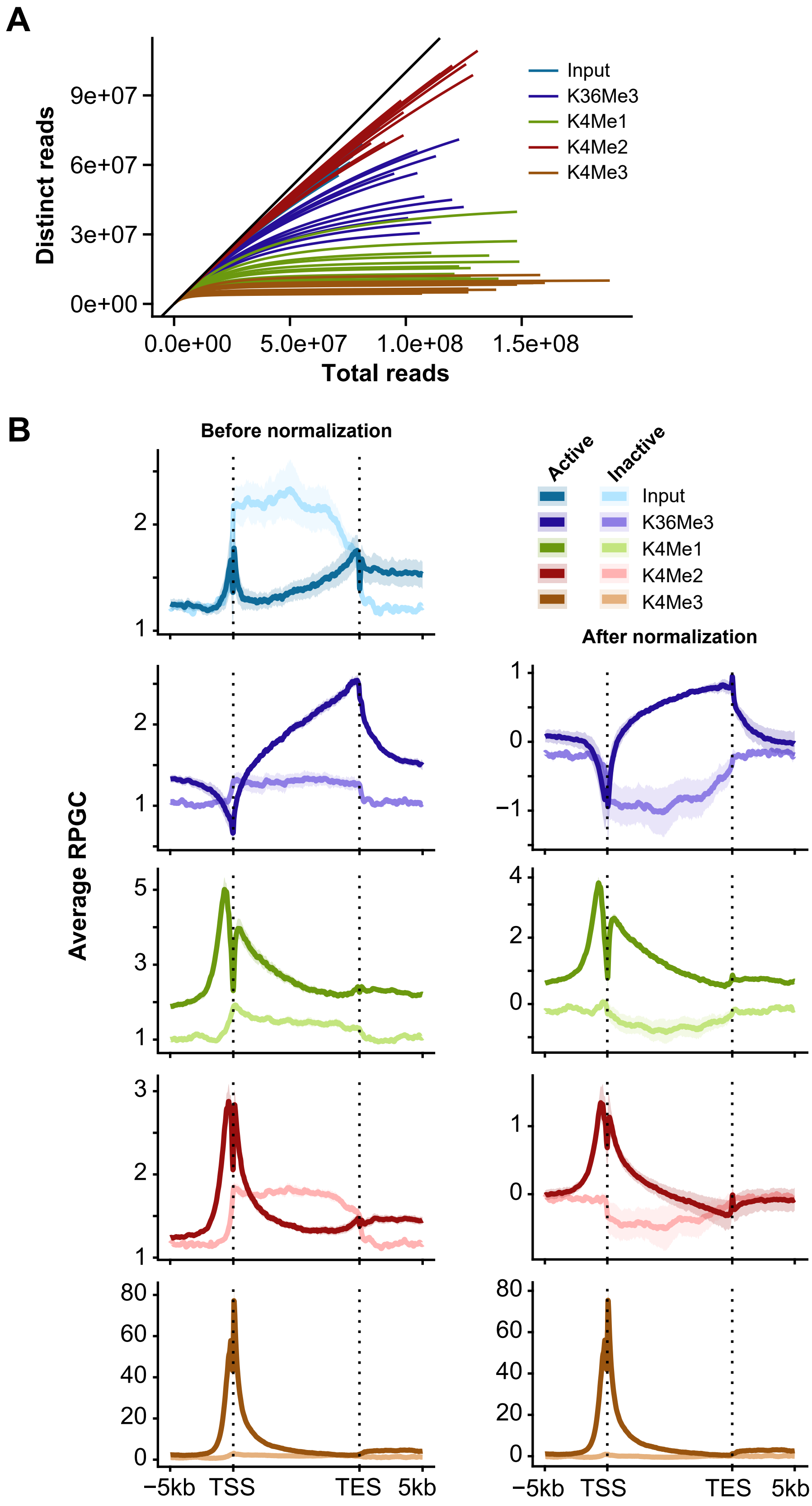
A
Distinct reads
9e+07
6e+07
3e+07
0e+00
0.0e+00
5.0e+07
1.0e+08
1.5e+08
Total reads
Input
K36Me3
K4Me1
K4Me2
K4Me3
B
Before normalization
After normalization
Active
Inactive
Input
K36Me3
K4Me1
K4Me2
K4Me3
Average RPGC
−5kb
TSS
TES
5kb

Figure S1

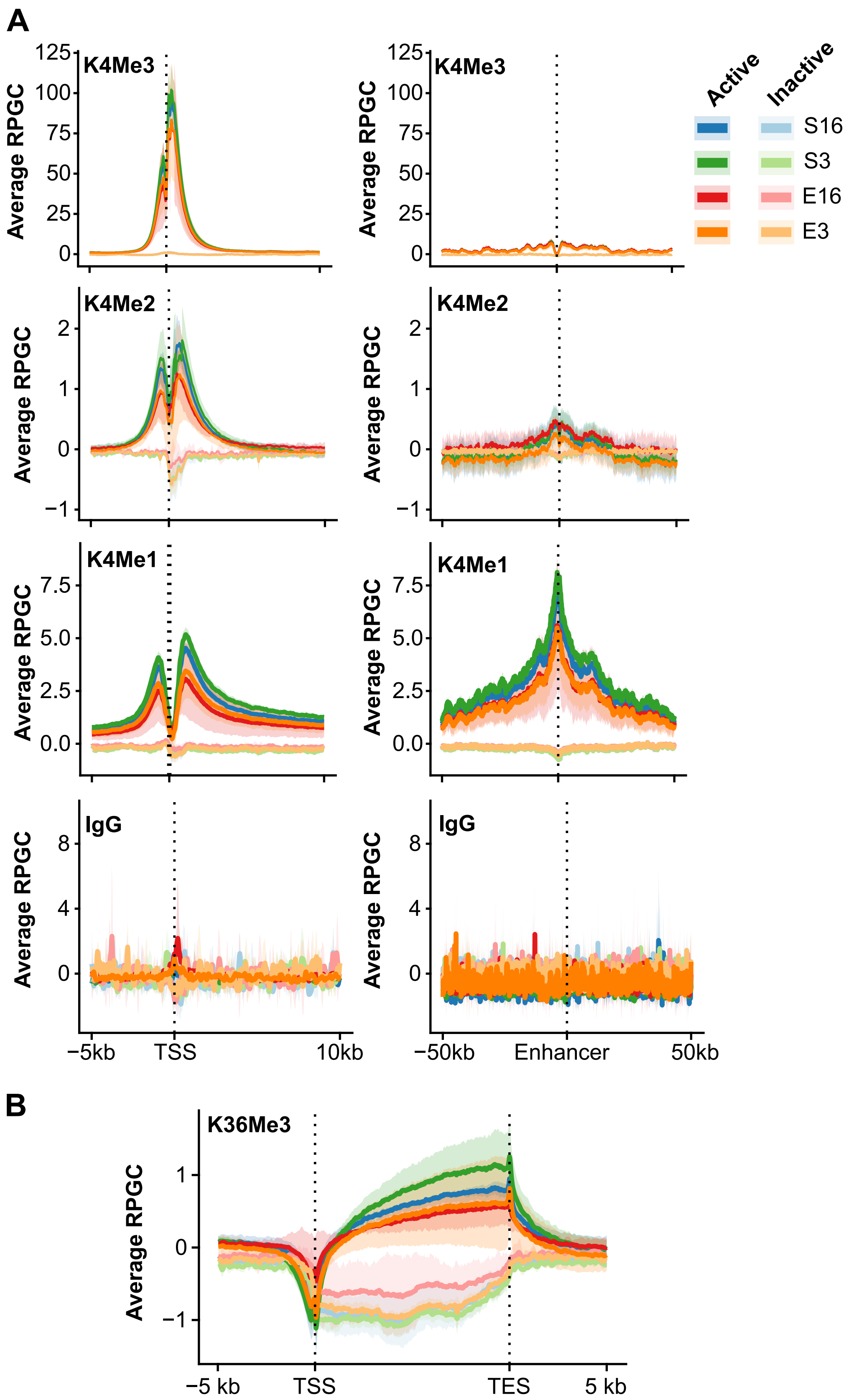

A
K4Me3
K4Me2
K4Me1
IgG
Average RPGC
Active
Inactive
S16
S3
E16
E3
−5kb
TSS
10kb
−50kb
Enhancer
50kb
B
K36Me3
−5 kb
TES
5 kb

Figure S2

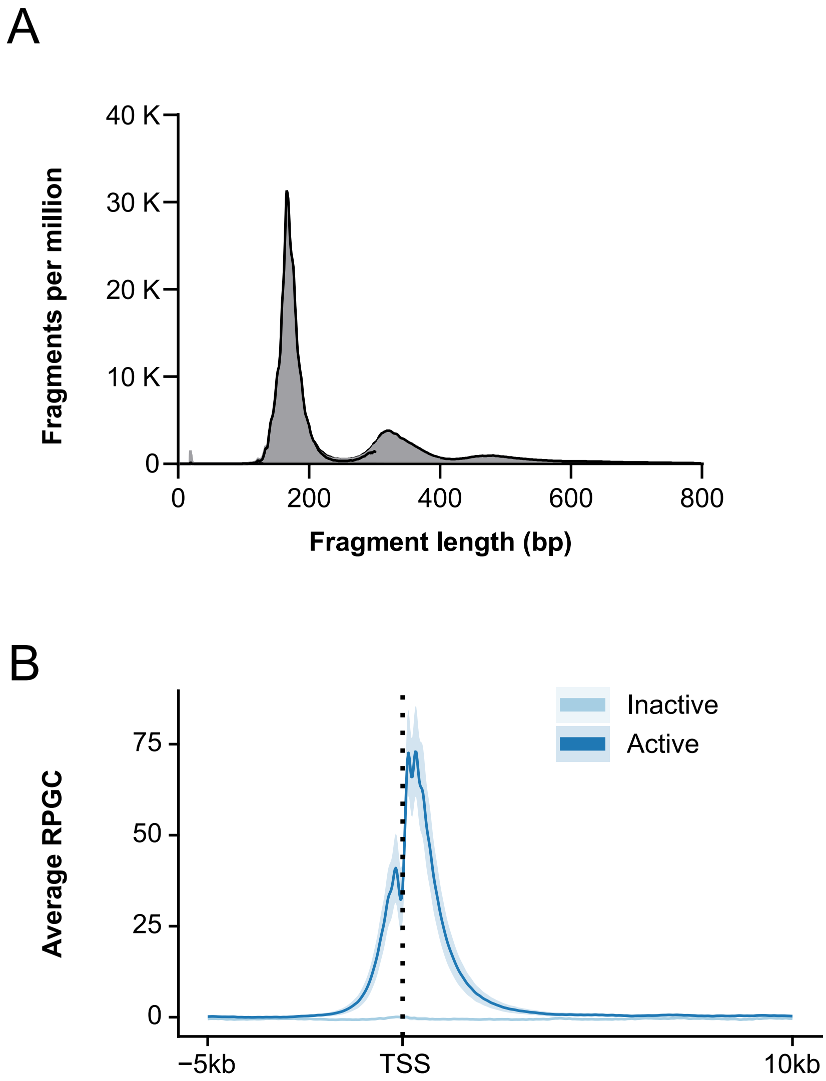
A
40 K
30 K
20 K
10 K
0
Fragments per million
0
200
400
600
800
Fragment length (bp)
B
Inactive
Active
75
50
25
0
Average RPGC
−5kb
TSS
10kb

Figure S3

A

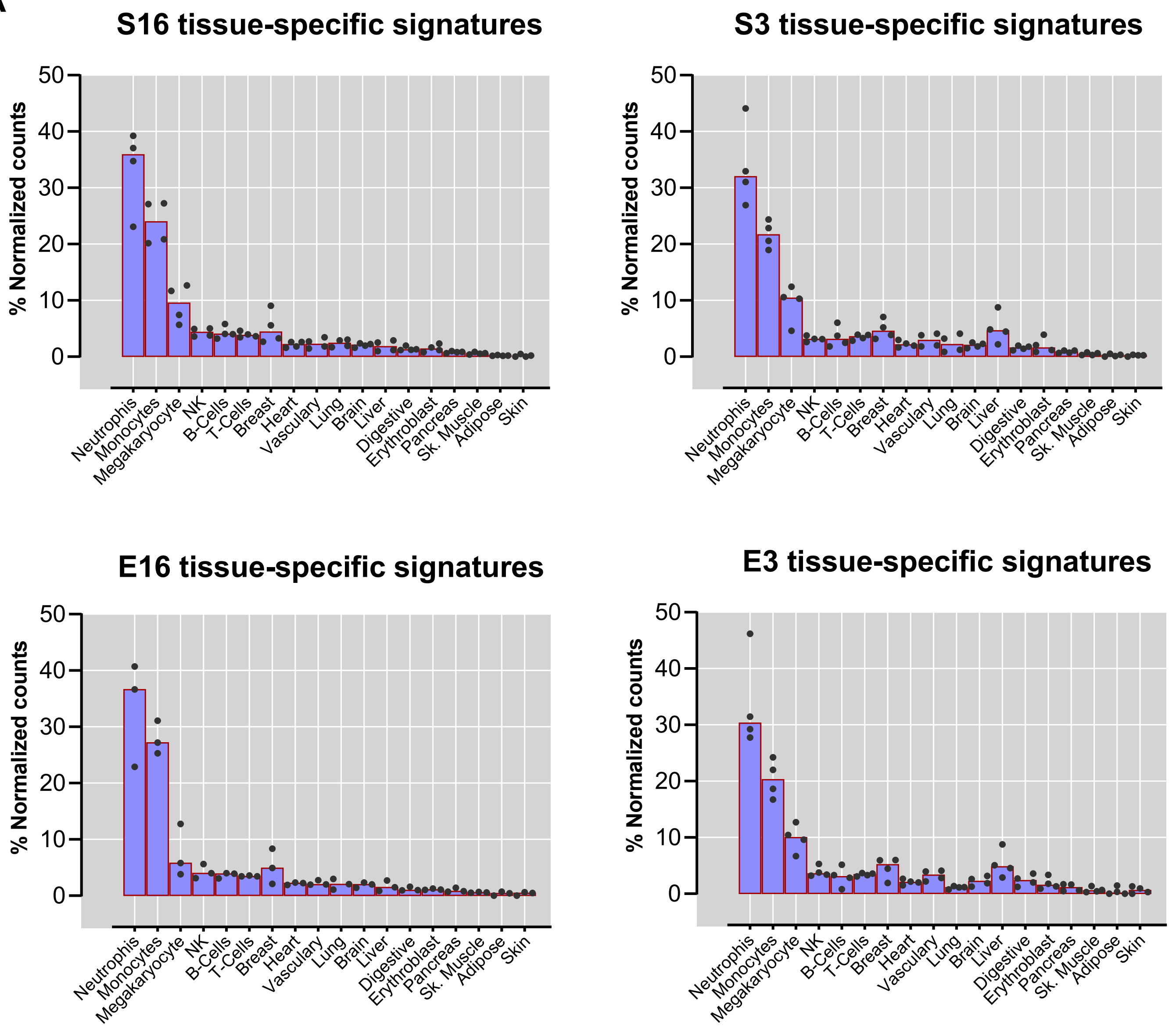

S16 tissue-specific signatures
S3 tissue-specific signatures
E16 tissue-specific signatures
E3 tissue-specific signatures
% Normalized counts
0
10
20
30
40
50
Neutrophis
Monocytes
Megakaryocyte
NK
B-Cells
T-Cells
Breast
Heart
Vascular
Lung
Brain
Liver
Digestive
Erythroblast
Pancreas
Sk. Muscle
Adipose
Skin

B

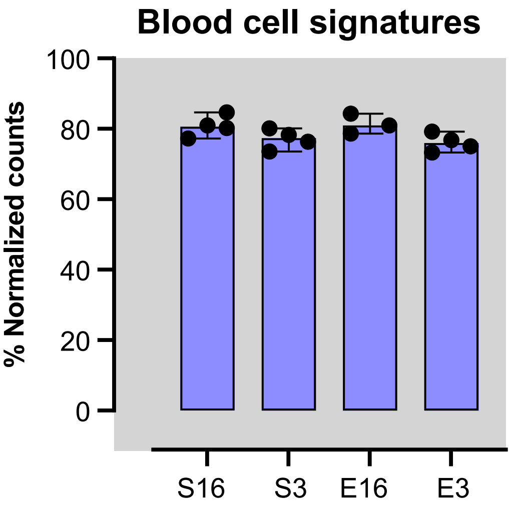

Blood cell signatures
% Normalized counts
0
20
40
60
80
100
S16
S3
E16
E3

Figure S4

bioRxiv preprint doi: https://doi.org/10.1101/2023.01.24.525414; this version posted January 25, 2023. The copyright holder for this preprint (which was not certified by peer review) is the author/funder, who has granted bioRxiv a license to display the preprint in perpetuity. It is made available under aCC-BY 4.0 International license.

Supplementary Table

Suppl. Table 1: Peaks, gene set with and without normalization for all four conditions

Suppl. Table 2: Housekeeping genes (also monocyte and neutrophil genes)

Suppl. Table 3: Number of differential genes by pathways
